# Assessing lumbar paraspinal muscle cross-sectional area and fat composition with T1 versus T2-weighted magnetic resonance imaging: Reliability and concurrent validity

**DOI:** 10.1371/journal.pone.0244633

**Published:** 2021-02-05

**Authors:** J. R. Cooley, J. J. Hebert, A. de Zoete, T. S. Jensen, P. R. Algra, P. Kjaer, B. F. Walker

**Affiliations:** 1 College of Science, Health, Engineering and Education, Murdoch University, Murdoch, Western Australia, Australia; 2 Faculty of Kinesiology, University of New Brunswick, Fredericton, New Brunswick, Canada; 3 Department of Health Sciences, Faculty of Science and Amsterdam Movement Science Research Institute, Vrije Universiteit, Amsterdam, The Netherlands; 4 Department of Diagnostic Imaging, Regional Hospital Silkeborg, Silkeborg, Denmark; 5 Department of Sports Science and Clinical Biomechanics, University of Southern Denmark, Odense M, Denmark; 6 Nordic Institute of Chiropractic and Clinical Biomechanics, Odense M, Denmark; 7 Noordwest Ziekenhuisgroep, Alkmaar, The Netherlands; 8 Health Sciences Research Centre, UCL University College, Odense M, Denmark; University of Southern California Keck School of Medicine, UNITED STATES

## Abstract

**Purpose:**

Studies using magnetic resonance imaging to assess lumbar multifidus cross-sectional area frequently utilize T1 or T2-weighted sequences, but seldom provide the rationale for their sequence choice. However, technical considerations between their acquisition protocols could impact on the ability to assess lumbar multifidus anatomy or its fat/muscle distinction. Our objectives were to examine the concurrent validity of lumbar multifidus morphology measures of T2 compared to T1-weighted sequences, and to assess the reliability of repeated lumbar multifidus measures.

**Methods:**

The lumbar multifidus total cross-sectional area of 45 patients was measured bilaterally at L4 and L5, with histogram analysis determining the muscle/fat threshold values per muscle. Images were later re-randomized and re-assessed for intra-rater reliability. Matched images were visually rated for consistency of outlining between both image sequences. Bland-Altman bias, limits of agreement, and plots were calculated for differences in total cross-sectional area and percentage fat between and within sequences, and intra-rater reliability analysed.

**Results:**

T1-weighted total cross-sectional area measures were systematically larger than T2 (0.2 cm^2^), with limits of agreement <±10% at both spinal levels. For percentage fat, no systematic bias occurred, but limits of agreement approached ±15%. Visually, muscle outlining was consistent between sequences, with substantial mismatches occurring in <5% of cases. Intra-rater reliability was excellent (ICC: 0.981–0.998); with bias and limits of agreement less than 1% and ±5%, respectively.

**Conclusion:**

Total cross-sectional area measures and outlining of muscle boundaries were consistent between sequences, and intra-rater reliability for total cross-sectional area and percentage fat was high indicating that either MRI sequence could be used interchangeably for this purpose. However, further studies comparing the accuracy of various methods for distinguishing fat from muscle are recommended.

## 1. Introduction

**Fig 1:**
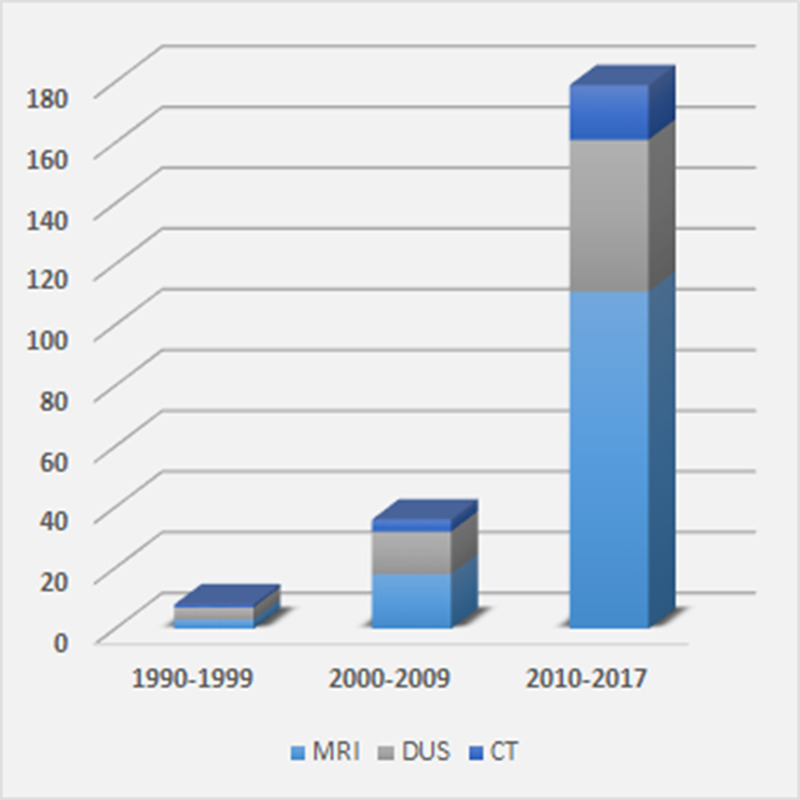
Number of imaging-related lumbar multifidus muscle research publications by decade: 1990–2017. MRI: magnetic resonance imaging; DUS: diagnostic ultrasound; CT: computed tomography [Databases: PubMed, Web of Science, Embase, SPORTDiscus, PEDro, CINAHL].

Over the last three decades, there has been a rapid increase in research interest regarding the role paraspinal muscles, and in particular the lumbopelvic stabilizing lumbar multifidi (LM), may play in relation to low back and leg pain. Most of this research has utilized diagnostic and functional imaging methods, including diagnostic ultrasound [1–4], computed tomography (CT) [5–7], and magnetic resonance imaging (MRI) [8–10]. From a previous systematic review [11], a comprehensive literature search from 1980–2017, focusing on imaging studies evaluating paraspinal muscles for various clinical, surgical, pathological, or anatomical reasons, identified an exponentially increasing use of advanced imaging to evaluate the paraspinal muscles (Fig 1). The majority of this research utilized MRI, particularly when looking at static evaluation of muscle.

Various MRI methods have been employed to assess the LM, ranging from the standard T1-weighted and T2-weighted spin-echo (spin echo) imaging sequences, to more sophisticated approaches such as functional [12, 13], opposed-phase [14], or chemical-shift MR [15], and MR spectroscopy [16]. However, spin echo (including fast/turbo spin echo) sequences are the most common methods used for MR imaging of the spine [17]. Studies incorporating spin echo imaging have utilised either T1-weighted [9, 18–21] or T2-weighted [8, 10, 22–25] sequences, but seldom provided the rationale for their sequence choice. There are, however, important technical considerations between T1 and T2-weighted sequences that could affect their ability to assess the anatomy or fat/muscle distinction within the LM.

Traditionally, T1-weighted sequences are described as providing greater anatomical detail than T2-weighted sequences (as T2-weighted sequences are more susceptible to motion artifact and lower signal-to-noise ratios) and better distinction between fat and fluid signal, due to the different T1 relaxation times of these two tissue types. Conversely, due to the longer T2 relaxation times for fat and fluid, the signals for these two tissues can both be high on T2-weighted sequences. Since muscle signal tends to be comparatively lower on T2 than on T1-weighted sequences, the signal difference between fat and muscle may be naturally greater on T2-weighted sequences [17, 26].

The question is whether these inherent differences are sufficient to negate our ability to apply these two sequences interchangeably, or even to compare outcomes between the two sequences. Suh et al. [27] compared the reliability of histographic analysis for T1 and T2-weighted sequences and found equivalent intra- and inter-rater reliability. However, this study did not include comparisons of muscle outlines, cross-sectional area (CSA) measures, or histographic outcomes between sequences.

While early evidence suggests that T1 and T2-weighted sequences are interchangeable for measuring paraspinal muscle morphology and atrophy, based on our literature search the validity of this assumption has not been previously tested. Therefore, the primary study objective was to examine the concurrent validity of LM morphology measures acquired from T2-weighted MR sequences compared to matched T1-weighted sequences. The secondary objective was to assess the intra-rater reliability of repeated LM measures for both T1 and T2-weighted imaging sequences.

## 2. Methods

Images accessed for this project were acquired within a general Dutch hospital population during 2009–2010. Authorization to access and evaluate images of the lumbar spine from this population, de-identified for all patient information, was provided by the head of the radiology department and the medical ethical committee of the Medical Centre Alkmaar, The Netherlands. Overall project approval was received from the Human Research and Ethics Committee at Murdoch University (approval: 2013/145]. As the images were fully de-identified, no patient demographics or clinical details were available for analysis.

### 2.1 Imaging parameters

MR images were acquired on 1.5T Siemens Symphony and Espree scanners, using the following parameters: T1 (TR/TE 411-610/12 msec, flip angle 150°) and T2-weighted (TR/TE 3230-5630/88-104 msec, flip angle 170°) axial turbo spin echo images; 4mm slice thickness; image resolution: 256 x 256 (T1), 320 x 320 (T2). Manual image angulation through the abnormal disc was made on the T1-weighted sequence, then copied for T2 image acquisition. To ensure muscle anatomy was directly matched across selected sequences, the location of axial slices was matched between the T1 and T2-weighted sequences by cross-referencing with the sagittal slices and confirming that the table and image slice location protocols were identical. Sequences that could not be precisely matched were excluded from analysis.

### 2.2 Image selection

Based on sample size calculations for agreement studies (α = 0.05; β = 0.90; *k* = 0) [28], 45 cases were required for analysis. These were randomly selected from a pool of 100 non-surgical MRI cases allocated for use by the Medical Centre, using a random number generator [29]. As the two imaging sequences of each patient were only being directly compared to each other, neither the presence or absence of surrounding pathology, nor the type of pathologies present were used for inclusion/exclusion purposes. Cases were included as long as they were of sufficient quality and scope to demonstrate the LM at L4/5 (L4) and L5/S1 (L5) bilaterally on both sequences, and did not demonstrate primary paraspinal muscle disease that would affect comparison (e.g., diffuse muscle edema). A total of 51 cases were randomly selected and reviewed, with exclusion of six cases which either did not demonstrate the required anatomical landmarks (3), demonstrated quality issues significant enough to affect measurement accuracy (e.g., abnormal alignment, severe pathology) (2), or failed to align the slice levels between the two sequences (1). The 45 cases included were sub-divided by spinal level (L4 and L5) and imaging sequence (T1 and T2) into 180 images and assessed bilaterally.

Image slice selection for each spinal level was based on the image that best demonstrated the following anatomical landmarks bilaterally: facet joints / articular processes, laminae, spinous process, and the lateral border of the LM on the same slice, as determined by the lead examiner (JC). Although this approach resulted in some between-case variation in slice levels, it did ensure the slices with the clearest LM boundaries were included. Once selected, each image was randomly assigned and encoded with a sequential image number by an assistant not associated with the project. This ensured the examiners were blinded to the randomization process and that the individual cases, spinal levels, and imaging sequences were assessed randomly. To undertake a second round of measurements, all images were re-randomized as above, with only the new image code included on the images.

### 2.3 Muscle morphology measurement procedures

The images selected were used to quantify muscle area versus fatty infiltrated tissue of the LM on T1 and T2-weighted sequences. Measures of LM morphology included: total CSA; total muscle (i.e., fat-free) CSA; and, total fat CSA. Measurement procedures were undertaken in a three-step process (described below) by the lead examiner (JC), who had over 30 years of experience in MRI interpretation as well as previous experience using sliceOmatic software.

To perform the measurements, we utilized sliceOmatic v5.0.7d [TomoVision, Magog, Canada]; it compared favourably in comparison studies with several of the above programs for adipose tissue assessment on MRI [19], and has been used extensively in adipose tissue and muscle quantification analysis research throughout the body [30] and specifically for evaluating cross-sectional LM morphology on MRI [20, 31]. This system allows for outlining muscle CSA and specific quantification of the fat and muscle tissue, including inbuilt calculation protocols which automatically adjusted for the different matrix sizes between the MR sequences used.

#### 2.3.1. Determining the muscle/fat transition value

For this study, we needed to account for variations between signal intensity and image acquisition size to compare different spin echo sequences, as well as considering the variations in image intensity from superficial to deeper structures, or from side to side, that can be present within an image. To identify a threshold value between muscle and fat also requires accounting for the fact that as muscle degrades towards fat it may do so gradually rather than fully, such that a broad grey-scale transition is present on the image. To attempt to account for each of the above variables, a protocol was developed using a histographic threshold analysis procedure, as this was considered to be the most efficient and consistent method to apply.

**Fig 2:**
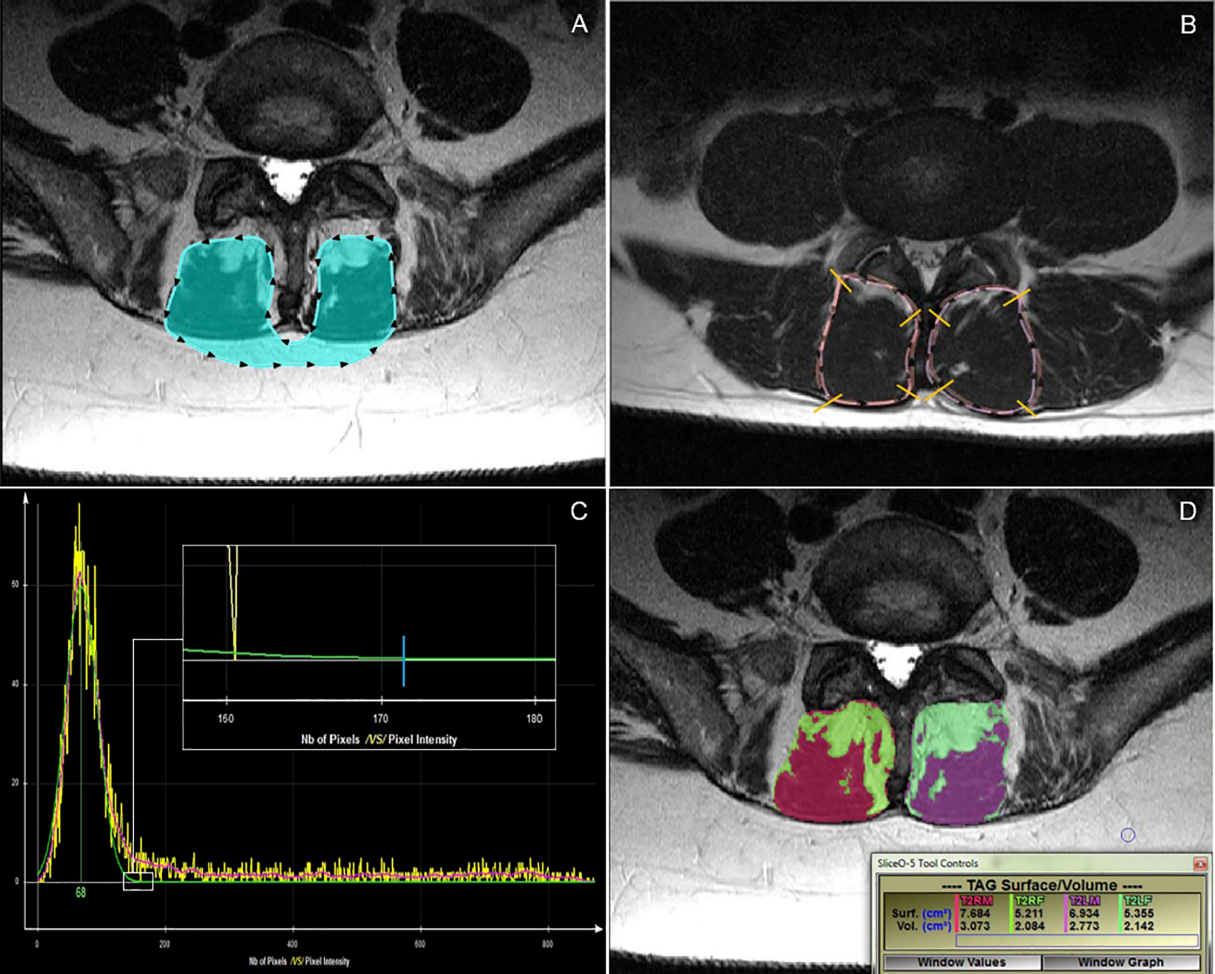
Different measurement procedure examples. **A:** Histogram outlining process. **B:** Histogram from A. Small white box indicates the region of intersection of the green histogram line with the X-axis at “0”. Note: to identify the point at which the histogram curve contacted the X-axis to the nearest value of 10, one must zoom in on the histogram within the sliceOmatic program. The inset box shows the point of intersection of the green histogram line with the X-axis, indicated by the blue line. In this example, the cut-off point would be 170. **C:** Overlapped T1-weighted and T2-weighted total cross-sectional area outlines for visual comparison. **D:** Fat (bright green/pale green) and muscle (red/purple) tissue highlighted, with related measurement outcomes [surf. measures (cm^2^) apply].

To determine the muscle/fat threshold value to apply bilaterally across the full depth of each muscle, the lead examiner acquired an initial histogram for each image by first outlining both multifidus muscles (connected via the subcutaneous fat but excluding any vertebral structures–see Fig 2A and 2B). The threshold was then determined by identifying the point at which the histogram curve intersected the X-axis to the nearest value of ten (see inset within Fig 2B). As this was a new cut-off determination method, the mean of two measures was acquired. The initial outline was deleted and a second outline acquired as described above, with the new intersecting value recorded. The average of these two values was inputted into a spreadsheet as the muscle/fat segmentation threshold.

For 25 images (14%), there was an insufficient amount of lean muscle mass on one or both sides of the image to acquire a valid histogram reading. In those instances, a visual estimation of the transition value between muscle and fat was determined by moving the cursor over pixels of muscles on each side and selecting a grey level image value that the examiner felt best represented the transition threshold. Although this process introduced a subjective element into the procedure, this scenario reflects clinical practice and represents a pragmatic solution to the interpretation of challenging images.

#### 2.3.2. Total CSA outlining procedure

Left and right multifidus outlines were individually traced with a computer mouse to create regions of interest corresponding to the cross-section of the muscle at that spinal level. For each measurement, the entire muscle boundary was manually outlined up to, but excluding, the cortical margins of the vertebral arch and supraspinous ligament medially and anteriorly, and posteriorly up to, but excluding, the superficial fascia. The clearest evidence of a fat/fascial boundary between the LM and erector spinae was used for the lateral margin. All muscle and fat within these boundaries were included. These measurement parameters accord with recent recommendations for assessing paraspinal muscle morphology [11, 15]. A detailed description of the outlining parameters applied, including methods for addressing variations from the “normal” boundary appearances, can be found in S1 File.

To assess qualitatively the similarity of anatomical outlines between imaging sequences, a snapshot of the initial outlined image was saved for later comparison of the muscle outlines between imaging sequences. Once all CSA measures were completed, the matching images between each sequence were overlaid (by making one image partially transparent), and the muscle boundaries divided into approximate quadrants. Screen magnification was set at 200%, and each quadrant’s outline between images rated as 0 = perfect/near perfect; 1 = mild mismatch; or, 2 = significant mismatch. Each muscle quadrant was rated individually and separately by two different examiners (JC, ADZ) for consistency of anatomical outlining between sequences (see Fig 2C). The protocols used to determine CSA outlining consistency were tested on five cases by each examiner, revised for clarity by consensus, and then performed on all cases. The final protocols used, including the criteria for qualitative agreement, can be found in S2 File. Once all ratings were initially completed, a follow-up consensus meeting was held and any discrepancies between examiner ratings discussed to reach final agreement on each rating.

#### 2.3.3. CSA measurements

Fat and muscle tissues were color-tagged by side and by imaging sequence for assigning measurement values (e.g., T2 right muscle = red; T2 left muscle = purple). The right and left-sided muscle outlines were then filled in with their corresponding color tag, creating total area and tissue-specific cross-sectional measurements that were exported to a spreadsheet for later analysis (see Fig 2D). SliceOmatic has the capacity for multiple images to be opened simultaneously, which allowed for assessment of images in groups of five. Once all images in a group were measured and the data exported, the outlines for each image were deleted. Those five images were then randomly reassessed by the same examiner and the measurement data exported. The means of these two measurements were used to analyse the CSA.

#### 2.3.4. Intra-rater analysis

To assess intra-rater reliability of the CSA measures, all 180 images were re-randomized and recoded, histographic analysis repeated, and muscles measures preformed bilaterally by JC at L4 and L5. To provide a period of time to reduce the likelihood of memory carryover, this phase started three weeks after all initial measurements had been completed.

### 2.4. Statistical analyses

Cross-sectional area measurements were recorded by level, side, and imaging sequence. Data were checked for non-plausible entries. Bland-Altman (BA) analysis (bias and limits of agreement (with 95% CIs)) and plots were calculated to compare T1 with T2-weighted outcomes for total CSA and percentage fat CSA. As percentage muscle CSA was merely the inverse result of percentage fat CSA, this measure was not reported.

While understanding the potentially arbitrary nature of establishing limits of agreement (LOA) for this study, an *a priori* range for acceptable variations in LOA of ±10% was set, based on previous studies on differences in multifidus CSA between symptomatic and normal/asymptomatic low back pain subjects [20, 32–36]. To apply this threshold to CSA values, the overall means for total CSA at L4 and L5 were calculated (based on the average of the means between the first and second measures of both sequences), with a mean total CSA at L4 of 10.0 cm^2^, and at L5, 10.8 cm^2^. For consistency of interpretation, the LOA 10% variability threshold for total CSA was set at ±1.0 cm^2^ for both spinal levels.

For the second round of measures, CSA was recorded by level and side, then the muscle CSA, fat CSA, total CSA, and percentage fat CSA quantitatively analysed against the initial measurements using two-way mixed effects, absolute agreement, single-rater intraclass correlation coefficients (ICC (3,1)); intra-rater ICC values greater than 0.90 were considered excellent [37]. Standard error of measurement and minimal detectable difference [1.96 x SQRT(2) x SEM] were determined.

To look more precisely at the distribution of any measurement variability relating to total CSA, percentage fat CSA and percentage muscle CSA, BA bias and 95% LOA statistics and plots were calculated for T1 and T2-weighted intra-rater measures. The *a priori* LOA threshold of ±10% (see above) was applied.

As there was a minimal difference in outcomes between sides for all analyses, right and left-sided outcomes were assessed together; agreement and reliability outcomes were reported bilaterally. The ICCs were calculated using SPSS v24 [IBM, Illinois, USA], while bias and LOA were calculated with STATA 15.1 [StataCorp LLC, Texas, USA] (for STATA coding, see S3 File).

## 3. Results

We included data from 45 participants (age and sex data excluded from cases), totalling 360 individual muscles analysed. The mean (±SD) total CSA at L4 was 10.06 (±2.06) cm^2^ (range: 5.98–17.23 cm^2^) on T1-weighted sequences and 9.84 (±2.07) cm^2^ (range: 5.80–16.65 cm^2^) on T2-weighted sequences; at L5, 10.92 (±1.84) cm^2^ (range: 7.29–15.32 cm^2^) on T1-weighted and 10.71 (±1.91) cm^2^ (range: 7.24–15.47 cm^2^) on T2-weighted sequences.

### 3.1 Levels of agreement between imaging sequences

**Fig 3:**
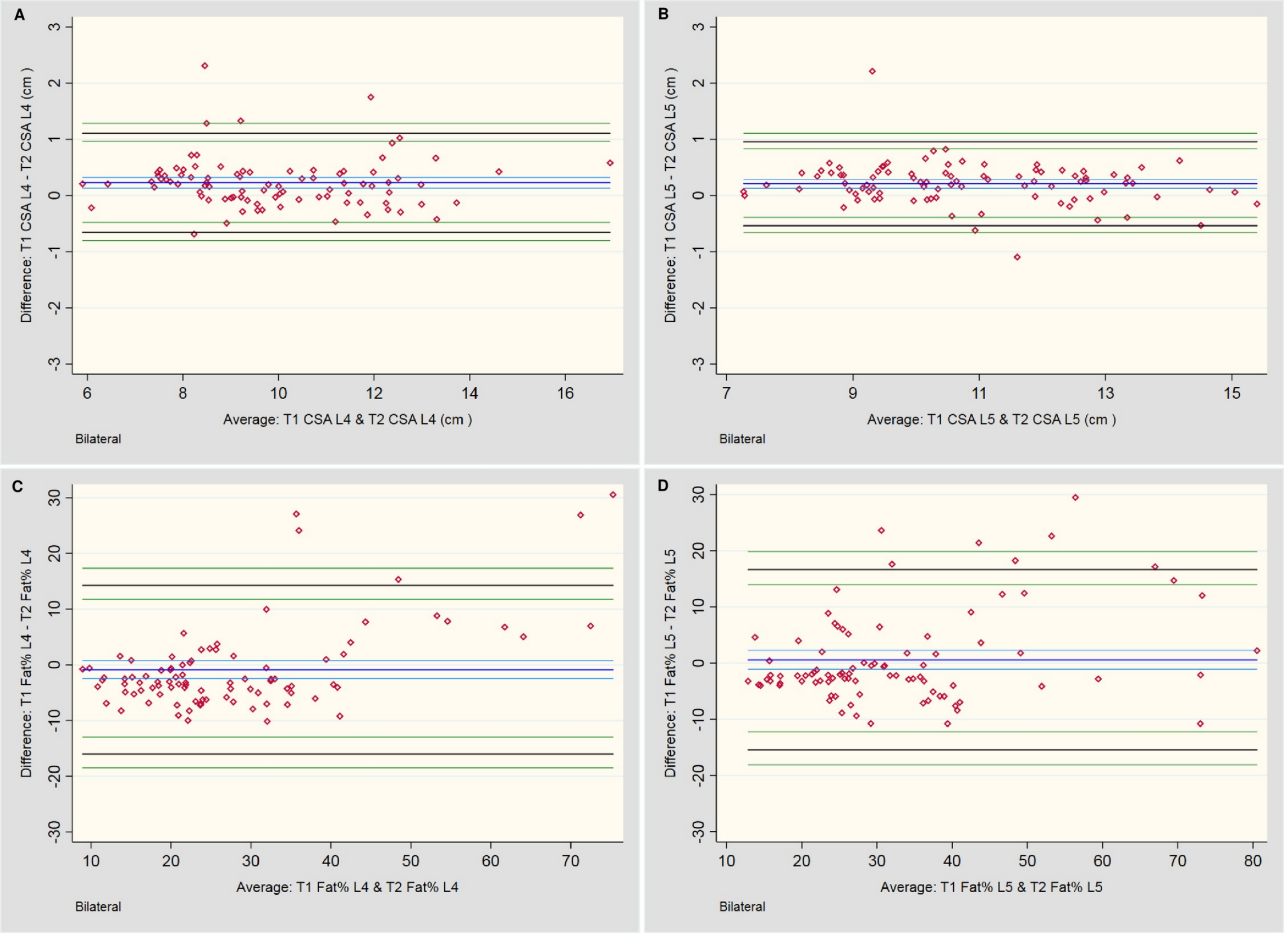
Bland-Altman plots for between-sequence measures. **A & B:** Total cross-sectional area (CSA) measures of the lumbar multifidi bilaterally at L4 and L5, respectively. **C & D:** Total fat area as a percentage of Total CSA (Fat %) bilaterally at L4 and L5, respectively.

The statistical outcomes and BA plots are provided in Table 1 and Fig 3, respectively. For total CSA measurements at L4 and L5, T1-weighted sequences systematically measured 0.2 cm^2^ larger than T2, although this would be an unimportant difference during practical application. Even with the small number of values outside the LOA range, the distribution of differences of the mean total CSA for the LM at both L4 and L5 appears relatively consistent across all measurement averages, falling within ±10%. However, analysis of fat as a percentage of total CSA was less consistent. Although no systematic bias was noted between the two imaging sequences, the LOA for percentage fat approached ±15% overall.

**Table 1:** Bland-Altman analysis: T1 and T2-weighted measures for total cross-sectional area (TCSA) and percentage fat (Fat %).

Bilateral (CSA)	N	Bias [cm^2^]	[95% CI]	Upper LOA	[95% CI]	Lower LOA	[95% CI]	Bias (Range) [cm^2^]	Magnitude (Range) [cm^2^]
T1 vs T2 TCSA L4	90	0.23	[0.13, 0.32]	1.11	[0.96, 1.29]	-0.66	[-0.48, -0.80]	-0.69, 2.31	5.90, 16.94
T1 vs T2 TCSA L5	90	0.21	[0.13, 0.29]	0.96	[0.83, 1.11]	-0.54	[-0.39, -0.66]	-1.10, 2.21	7.27, 15.40
Bilateral (Fat % of CSA)*	N	Bias [%]	[95% CI]	Upper LOA	[95% CI]	Lower LOA	[95% CI]	Bias (Range) [cm^2^]	Magnitude (Range) [cm^2^]
T1 vs T2 Fat % L4	90	-0.86	[-2.46, 0.74]	14.29	[11.78, 17.35]	-16.02	[-12.96, -18.53]	-10.14, 30.56	8.94, 75.29
T1 vs T2 Fat % L5	90	0.59	[-1.10, 2.29]	16.64	[13.99, 19.88]	-15.46	[-12.22, -18.12]	-10.77, 29.50	12.86, 80.53

CI: confidence interval; LOA: limits of agreement.

*All % muscle results were inversely identical to % fat results so were not included.

### 3.2. Muscle outlining consistency

Visual analysis of muscle outlining demonstrated perfect or nearly perfect consistency between sequences, at each level and bilaterally, in 83% of cases (Table 2). Conversely, significant outlining mismatches only occurred bilaterally along 4.8% of the muscle boundaries, being twice as common at L4, and much more likely to involve the anterior or lateral margins (80%).

**Table 2:** Ratings for visual assessment of muscle outline.

	Right-sided ratings = 2*	Right-sided ratings = 1	Right-sided ratings = 0	Totals	Left-sided ratings = 2	Left-sided ratings = 1	Left-sided ratings = 0	Totals
**L4**								
Anterior	3	9	33	45	3	8	34	45
Medial	1	3	41	45	0	4	41	45
Posterior	1	6	38	45	3	4	38	45
Lateral	5	6	34	45	7	2	36	45
Totals (%)	10 (6)	24 (13)	146 (81)	180	13 (7)	18 (10)	149 (83)	180
**L5**								
Anterior	2	6	37	45	2	11	32	45
Medial	0	7	38	45	1	1	43	45
Posterior	0	1	44	45	1	1	43	45
Lateral	3	11	31	45	3	6	36	45
Totals (%)	5 (3)	25 (14)	150 (83)	180	7 (4)	19 (11)	154 (86)	180

* Ratings: 2 = significant mismatch between images; 1 = mild mismatch between images; 0 = perfect/ near perfect overlap between images

Regarding the distribution of cases requiring consensus for agreement of ratings (Table 3), the spinal levels and sides were relatively equal; however, the anterior and lateral margins were more than twice as likely to require discussion to reach consensus. This corresponds with the higher levels of outlining variations at the anterior and lateral boundaries between imaging sequences noted in the visual analysis.

**Table 3:** Cases requiring consensus between raters for visual assessment of muscle outline.

	L4		L5		Totals	%*
	Right	Left	Right	Left		
Anterior	8	6	8	9	31	*17*
Medial	3	2	3	3	11	*6*
Posterior	4	5	2	1	12	*7*
Lateral	6	6	10	7	29	*16*
Totals	21	19	23	20	-	-
% totals*	*12*	*11*	*13*	*11*	-	-

* Each % total is based on Total/180 rating pairs

### 3.3. Intra-rater reliability, bias, and limits of agreement

Reliability was excellent for all CSA measures, with ICC values ranging from 0.981–0.998 (Table 4). Neither location (L4 or L5) nor sequence (T1 or T2-weighted) resulted in any important reduction in reliability.

**Table 4:** Intra-rater reliability for measurement of cross-sectional area (CSA).

Tissue type (level)	N	Mean (SD)(cm^2^) 1^st^ measures	Mean (SD)(cm^2^) 2^nd^ measures	ICC (95% CI)*	SEM (cm^2^)	MDD (cm^2^)
**T1**						
Fat CSA (L4)	90	2.72 (1.68)	2.68 (1.64)	0.998 (0.995–0.998)	0.074	0.206
Fat CSA (L5)	90	3.56 (1.74)	3.51 (1.77)	0.996 (0.993–0.998)	0.111	0.308
Muscle CSA (L4)	90	7.34 (2.36)	7.36 (2.31)	0.993 (0.989–0.995)	0.195	0.541
Muscle CSA (L5)	90	7.36 (2.36)	7.39 (2.38)	0.995 (0.993–0.997)	0.168	0.465
Total CSA (L4)	90	10.06 (2.06)	10.04 (2.00)	0.988 (0.983–0.992)	0.222	0.615
Total CSA (L5)	90	10.92 (1.84)	10.90 (1.87)	0.990 (0.985–0.993)	0.186	0.514
**T2**						
Fat CSA (L4)	90	2.77 (1.29)	2.75 (1.28)	0.994 (0.990–0.996)	0.100	0.276
Fat CSA (L5)	90	3.46 (1.54)	3.45 (1.54)	0.988 (0.982–0.992)	0.169	0.468
Muscle CSA (L4)	90	7.07 (2.01)	7.11 (2.00)	0.990 (0.985–0.993)	0.200	0.555
Muscle CSA (L5)	90	7.24 (2.06)	7.19 (1.98)	0.981 (0.971–0.987)	0.278	0.772
Total CSA (L4)	90	9.84 (2.07)	9.86 (2.04)	0.989 (0.983–0.993)	0.216	0.598
Total CSA (L5)	90	10.71 (1.91)	10.64 (1.87)	0.984 (0.976–0.989)	0.239	0.663
Tissue type (level)	N	Mean (SD)(%) 1^st^ measures	Mean (SD)(%) 2^nd^ measures	ICC (95% CI)*	SEM (%)	MDD (%)
T1**						
Fat/CSA% (L4)	90	27.43 (16.55)	27.03 (16.26)	0.998 (0.996–0.999)	0.733	2.033
Fat/CSA% (L5)	90	33.12 (16.63)	32.62 (16.82)	0.997 (0.995–0.998)	0.916	2.539
T2**						
Fat/CSA% (L4)	90	28.29 (12.14)	28.08 (12.05)	0.993 (0.989–0.995)	1.012	2.805
Fat/CSA% (L5)	90	32.52 (14.07)	32.45 (13.62)	0.986 (0.978–0.990)	1.639	4.246

ICC: intraclass correlation coefficient; SD: standard deviation; CI: confidence interval; SEM: standard error of measurement; MDD: minimal detectable difference.

* All ICC values significant at *p* = 0.000.

**Fig 4:**
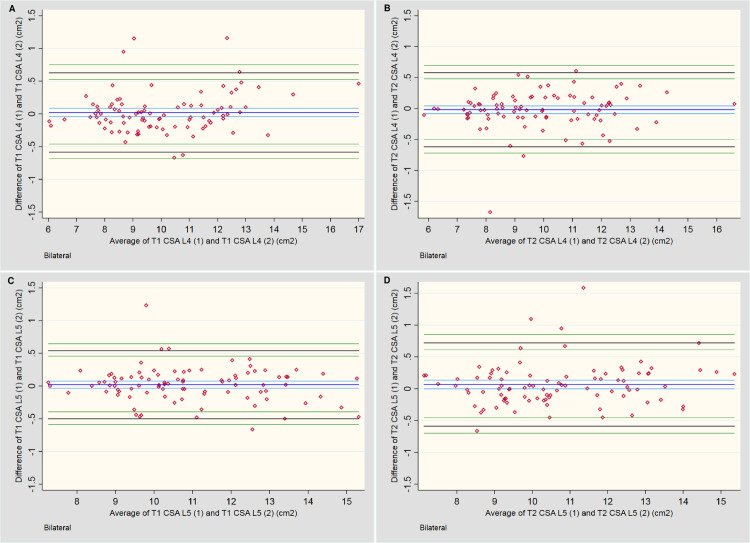
Bland-Altman plots for intra-rater assessment of total CSA. **A & B:** Measures of the lumbar multifidi at L4 for T1 and T2-weighted imaging, respectively. **C & D:** Measures of the lumbar multifidi at L5 for T1 and T2-weighted imaging, respectively.

Table 5 and Fig 4 provide summaries of the descriptive outcomes and BA plots, respectively, for the *total CSA*. The initial measures were slightly larger (0.1 cm^2^) than the second, but the distribution was generally consistent across the range of measurements. Any larger variations tended to occur in muscles with a smaller total CSA.

**Table 5:** Bland-Altman analysis: Intra-rater measurement of total cross-sectional area (TCSA) and percentage fat (Fat %).

Bilateral (CSA)	N	Bias [cm^2^]	[95% CI]	Upper LOA	[95% CI]	Lower LOA	[95% CI]	Bias (Range) [cm^2^]	Magnitude (Range) [cm^2^]
T1 vs T1 TCSA L4	90	0.02	[-0.04, 0.09]	0.63	[0.53, 0.75]	-0.58	[-0.46, -0.68]	-0.67, 1.16	6.03, 16.99
T1 vs T1 TCSA L5	90	0.02	[-0.03, 0.08]	0.54	[0.45, 0.65]	-0.50	[-0.40, -0.59]	-0.66, 1.23	7.29, 15.31
T2 vs T2 TCSA L4	90	-0.02	[-0.08, 0.04]	0.58	[0.48, 0.70]	-0.62	[-0.50, -0.72]	-1.67, 0.61	5.85, 16.61
T2 vs T2 TCSA L5	90	0.07	[-0.00, 0.14]	0.72	[0.61, 0.85]	-0.59	[-0.46, -0.70]	-0.66, 1.59	7.13, 15.36
Bilateral (Fat % of CSA)*	N	Bias [%]	[95% CI]	Upper LOA	[95% CI]	Lower LOA	[95% CI]	Bias (Range) [cm^2^]	Magnitude (Range) [cm^2^]
T1 vs T1 Fat % L4	90	0.40	[0.18, 0.62]	2.50	[2.15, 2.92]	-1.70	[-1.28, -2.05]	-2.12, 3.66	8.35, 88.99
T1 vs T1 Fat % L5	90	0.49	[0.24, 0.74]	2.82	[2.44, 3.29]	-1.84	[-1.37, -2.22]	-3.49, 4.03	10.78, 80.14
T2 vs T2 Fat % L4	90	0.21	[-0.09, 0.50]	2.99	[2.53, 3.55]	-2.58	[-2.01, -3.04]	-4.53, 5.05	9.45, 67.86
T2 vs T2 Fat % L5	90	0.07	[-0.42, 0.56]	4.70	[3.93, 5.64]	-4.56	[-3.63, -5.33]	-15.20, 4.37	11.60, 77.80

CI: confidence interval; LOA: limits of agreement.

* Muscle % results were merely inversely proportional to fat % results, so are not included.

**Fig 5:**
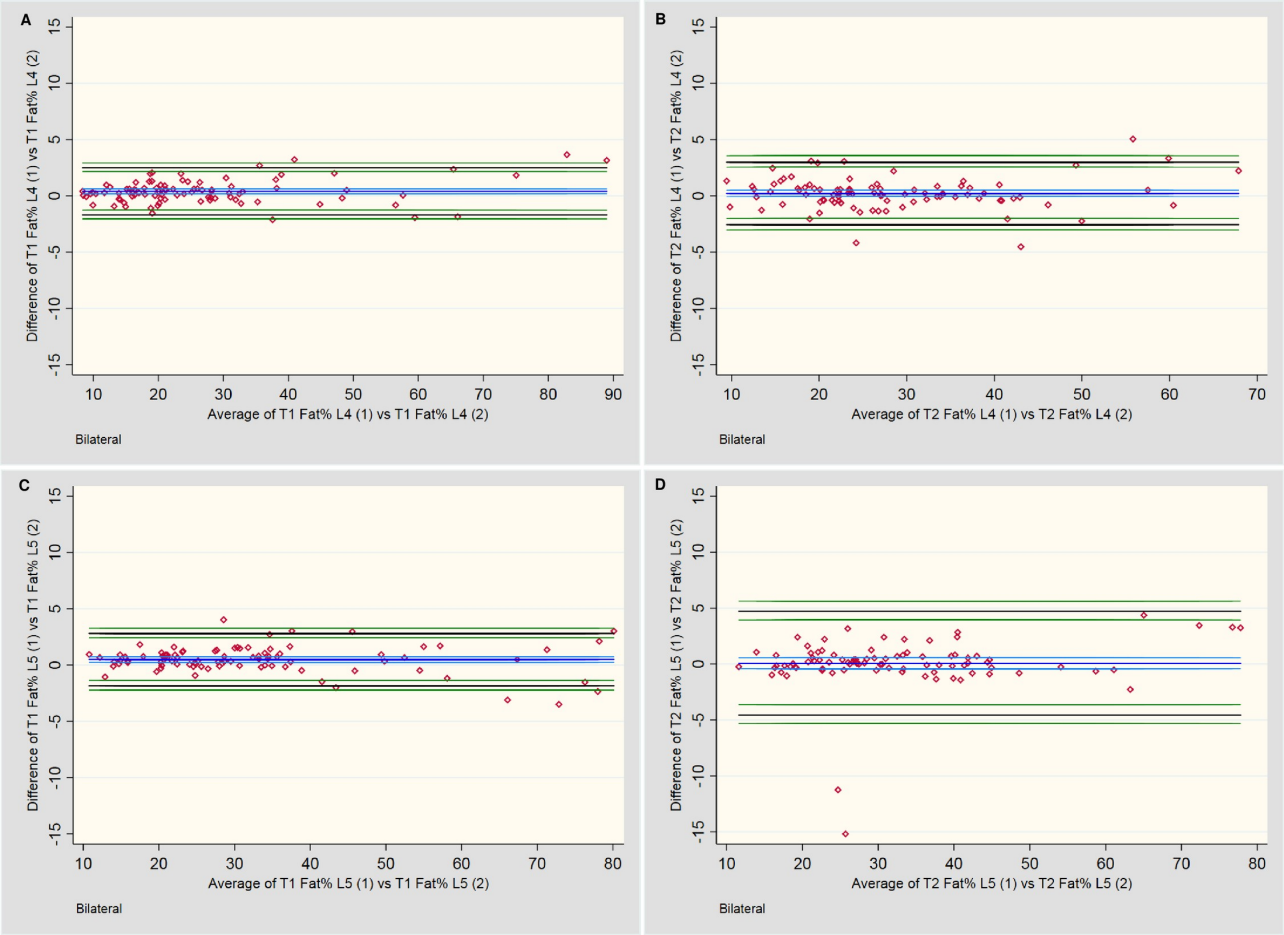
Bland-Altman plots for intra-rater assessment of fat percentage (Fat %) of total CSA. **A & B:** Measures of the lumbar multifidi Fat % at L4 for T1 and T2-weighted imaging, respectively. **C & D:** Measures of the lumbar multifidi Fat % at L5 for T1 and T2-weighted imaging, respectively.

Table 5 and Fig 5 provide the descriptive outcomes and BA plots, respectively, for the *percentage fat CSA*. A slight systematic bias was noted for two outcomes, with a tendency towards larger percentage outcomes for the first measures; however, this bias was less than 1.0% at either level and for either sequence. A mild increase in variability of measures occurred once ~60% fat was present. On the T2-weighted sequences at L5 (Fig 5D), two outcomes exceeded the 10% variability threshold between measures, which appears to have artificially increased the LOA compared to the other three plots. The remaining measures fell within ±5%.

## 4. Discussion

When considering the interchangeability of T1 and T2-weighted sequences to measure the lower lumbar multifidus muscles, the total CSA would appear to be consistent between sequences, but not necessarily when measuring the CSA of different tissue types (e.g., fat) within the muscle boundaries. Although no systematic bias was present between the two sequences when assessing the percentage of fat within the total CSA, the differences between sequences became more variable when less muscle was present.

Contributing factors for the increased variability in distinguishing muscle from fat between sequences may include: 1) for a small percentage of cases, muscle outlining was substantially different between sequences; however, this affected cases with ample healthy muscle tissue as well as reduced muscle tissue, so would seem to be a small contributor; 2) the ability of the software’s histogram tool to identify muscle and fat peaks when there were limited amounts of muscle was problematic, requiring visual estimation of the threshold values, which introduced potential for threshold value error between sequence; but cases assessed with the visual method only contributed to some of the outliers so this doesn’t account for all variability; 3) T1-weighted sequences may inherently have higher fat signal than T2-weighted sequences, which could have accentuated the differences between T1 and T2-weighted tissue signal as the fat percentage increased; conversely, muscles with severe atrophy secondary to chronic muscle edema may have been included, resulting in mild accentuation of T2-weighted versus T1-wieghted muscle signal differences in a small number of cases.

Neither the spinal level nor body side had a notable impact on any measurement outcomes. Additionally, as ~95% of muscle outlines showed minimal to no difference between sequences, any agreement that was found in the total CSA measurements was based on direct matching of muscle outlines, not fortuitously similar cross-sectional measures of incorrectly outlined muscles. This confirms that outlining of muscles can also be performed consistently on either MR sequence–although the following limitations should be considered.

When outlining the muscle boundaries, adequate visualization of landmarks is crucial for consistency. Two keys factors came into play in this regard: 1) the variability of anatomy between patients; 2) the variability of landmarks between MR sequences in the same patient. When considering “between patient” variability, the medial and posterior boundaries had relatively consistent margins to follow, with the spinous process and posterior fascial boundaries generally fully visible on every image. These two boundaries were the least likely to show a significant mismatch between sequences, or to require a consensus discussion to confirm an outline rating. For the anterior and lateral margins, this was not the case.

A protocol has been suggested to alleviate variations in outlining these margins [15], and we developed an additional protocol (see S1 File), which improved consistency; however, these accommodations are unable to address all potential variations in slice plane anatomy. Anteriorly, the laminar cortex may or may not be visible across the full margin, and the facet joint / articular process anatomy may be fully, or only partially, present; the presence of facet joint hypertrophy adds another layer of complexity.

Laterally, the margins between the multifidus and erector spinae muscles are often indistinct, particularly when the patient has less body fat to enhance the fascial boundaries. The upper and lower aspects of this margin are at times effectively invisible, with no adjacent reference points to assist. Each of these issues is likely to require the examiner to “estimate” the true boundaries.

**Fig 6:**
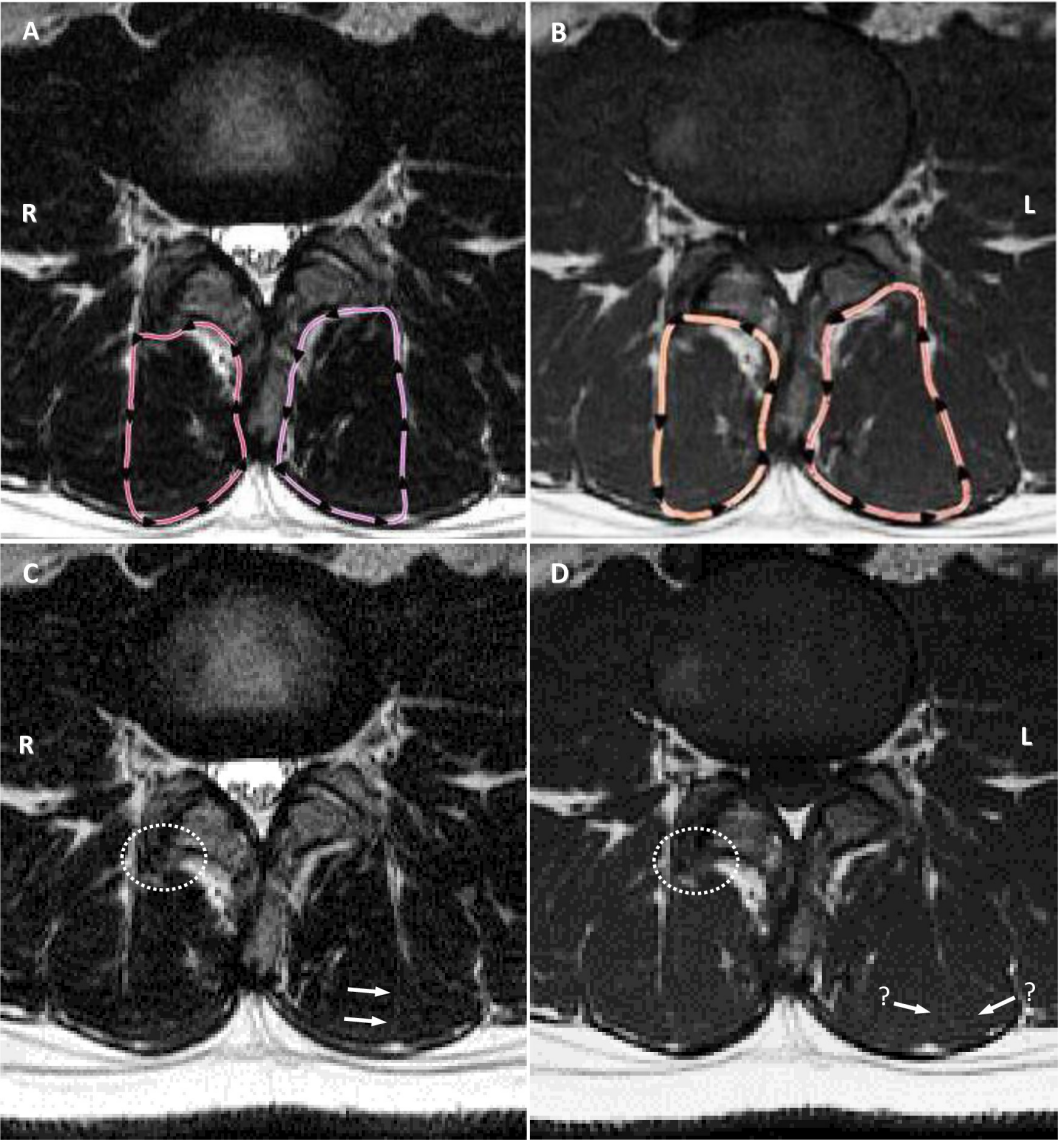
Examples of subtle but important variations in appearance of anatomy. **A & B:** T2-weighted and T1-weighted sequences with cross-sectional outlines. Note obvious differences in outlining adjacent to the facet joint on the right and lateral muscle margin on the left, both rated at 2 for mismatch. **C:** T2-weighted image (original). Arrows highlight the subtle fascial plane used by the examiner as the dividing line between multifidus and erector spinae muscle groups; white dotted circle highlights obvious bone hypertrophy from facet arthrosis, not included by the examiner. **D:** T1-weighted image (original). Arrows with “?” highlight the absence of a clear fascial plane, with two potential options open to the examiner–the outer option was used; white dotted circle highlights same region as image C, but the type of tissue is not as obvious, and was included as muscle on the T1 image.

When comparing the ability of T1 and T2-weighted sequences to assess the LM anatomy in the same patient, subtle variations in brightness or darkness of muscle boundary anatomy, difference in image matrix size (i.e., small differences in magnification when viewing the margins), and slight variations in slice location due to patient movement or breathing differences between slice acquisitions, may ultimately determine whether the muscle boundaries will be visible. This effect was most apparent at the anterior and lateral margins in a small number of cases in our study, due to the inherent challenges previously discussed. Fig 6 exemplifies these issues.

Intra-rater agreement was excellent and consistent for both imaging sequences, with the small variations that did occur within the total and percentage fat CSA unlikely to represent a clinically important difference for multifidus measures. This indicates that the challenges in identifying LM boundaries can be substantially overcome by using a standardized approach, with the appropriate protocols in place to address issues of poor boundary visualization. Two prior studies utilizing the sliceOmatic software to measure the multifidus muscle morphology reported similarly high intra-rater reliability, although these studies only reported an overall outcome from a single MRI sequence [20, 31]. Our study is the first to use sliceOmatic to directly assess intra-rater reliability measures of the LM across different imaging sequences.

Some limitations with this study were identified and accounted for where feasible. First, as the images were accessed from an existing database, there was no control as to how the images were acquired. Additionally, muscles in a variety of states of health, and images that did not always clearly demonstrate all muscle boundaries, were included. These variables required occasional compromises when selecting cases for inclusion, with slices containing clear demonstrations of muscle boundaries bilaterally, at two spinal levels, across both imaging sequences not always possible to obtain. This contributed to some of the inconsistency in outlining muscles in a small number of cases; however, this would have impacted both sequences approximately the same. While many of the consistency issues we experienced may have been prevented by including only those images with the best overall quality, it was decided that that approach would not provide a realistic comparison for application in the clinical setting. Second, the examiner could not be blinded to the imaging sequence during measurements, as this was evident on each image. There was, however, no apparent bias towards finding either sequence superior, so any negative effects should have been negligible. Third, using a histogram method to distinguish muscle from fat, although commonly used for this purpose, has inherent limitations in accuracy when one tissue type is mostly absent; this may be accentuated when adapting the process to compare different MR sequences. Alternative methods of distinguishing functional multifidus muscle from non-functional tissue (e.g., Beneck, Fortin [20, 23]) could be tested to see if this issue can be overcome. Fourth, only one examiner measured the CSA, which has the potential for measurement bias. This approach was used to efficiently address the primary aim of the study, being a basic comparison between the two imaging sequences rather than a more complex interrater reliability study. To help identify and/or reduce any bias, an experienced examiner used the means of two different measures on two different occasions, and an in-depth intra-rater analysis was implemented to look for areas of unreliability along the entire measurement spectrum. Where bias could not be adequately address (i.e., when assessing the outlining of muscle boundaries), a second examiner with no involvement in the measurement process and blinded to the measurement results was utilized. Finally, the establishment of a clinically relevant range for LOA needs to account for the inherent errors that occur with any manual measurement system. No comparable studies comparing the use of two spin echo sequences to measure multifidus muscle morphology were available to establish this range in an *a priori* manner, although it was deemed important to pre-determine this range. The potentially arbitrary nature of the value we established is acknowledged.

### 4.1 Conclusions

In this study, total CSA measures and the outlining of LM muscle boundaries were consistent between sequences, indicating there are no important concerns with using T1 or T2-weighted sequences interchangeably for this purpose with an experienced examiner. Intra-rater reliability in measuring total CSA and the percentage of fat or muscle within the total CSA was also high, confirming either MRI sequence could be used reliably by the same assessor. However, we found inconsistent identification of the functional muscle and/or fat area within the total muscle CSA, with a reduction in consistency of tissue-specific measurements as the fat percentage increased, particularly at L5. Using a histogram method to determine the muscle/fat threshold value could have potentially affected the accuracy of outcomes, and further studies comparing the accuracy of the various methods available for this purpose are recommended. The effect on agreement between sequences by multiple examiners of different levels of experience could also be undertaken.

## Supporting information

S1 File Criteria for outlining muscle boundaries for total CSA measures. (PDF)

S2 File T1/T2 qualitative (visual) muscle outlining comparison protocols. (PDF)

S3 File STATA coding formulas for Bland-Altman analysis. (PDF)

## Abbreviations / initializations

LM: lumbar multifidi/multifidus
CSA: cross-sectional area
