# Supplementary material for: Assessing lumbar paraspinal muscle cross-sectional area and fat composition with T1 versus T2-weighted magnetic resonance imaging: Reliability and concurrent validity
Source: PLoS One. 2021 Feb 5;16(2):e0244633. doi: 10.1371/journal.pone.0244633 (PMC7864460; doi:10.1371/journal.pone.0244633)
Supplement: S1 File — (PDF) [file pone.0244633.s001.pdf]

1. Medial border:
    - Outline along the outer margin of the cortex of the SP, transitioning to the lamina superiorly and the posteromedial fascial plane inferiorly.
    - Caveats:
        - If the SP does not reach the posteromedial fascial plane, follow the outer margin of the fascial plane separating the right and left muscles from the tip of the SP to the posteromedial fascial boundary.
        - If the SP does not reach the posteromedial fascial plane, and there is muscle (or ligament) posterior to the SP with no clear fascial separation, then follow the inner margins of the muscle on each side to the posterior fascial boundary (i.e., don't include this muscle).

2. Superior (anterior) border:
    - Outline along the outer margin of the cortex of the lamina, extending to the outer fascial muscle plane or the facet joint, whichever comes first [if to the facet joint, then extend horizontally if needed to the outer muscle plane].
    - Caveats:
        - If the full lamina is not obvious, extend anteriorly as needed to the visible laminar margin, as far as the cortical margin of the spinal canal (the image slice is probably at the edge of the lamina). If the entire laminar margin is indistinct, follow any fragments that best suggest a cortical edge.
        - If there is obvious facet hypertrophy, do not include it in the outline. If unclear whether bone or muscle, include it.
        - If there is no fascial plane visible at the lateral margin, go to the facet joint and then extend down or out to the nearest visible LM margin.
        - If no facet joint or fascial plane is present laterally, go to most lateral laminar/arch margin, then extend directly to nearest outer LM margin.

3. Lateral border:
    - Follow the lateral LM margin / clearest fascial plane separating the LM from the other ES muscles, to the posterolateral fascial plane.
    - Caveats:
        - If the delineation between LM and ES is unclear, extend along the margin that leads to the dimple (if visible) at the posterolateral fascial plane. If using the dimple, ensure it's between the LM and ES, not the two components of the ES.
        - If the delineation between LM and ES is unclear, there is no obvious dimple, and the appearance of the orientation of the muscle bundles is unclear, draw a vertical line connecting the last visible fascial plane between the two muscles to the posterolateral fascial plane.
        - If the muscle boundary is unclear and there is no dimple, but it is possible to see the difference in muscle fiber orientation, use this as the dividing line.

4. Inferior (posterior) border:
    - Outline along the inner margin of the posterior fascial plane from lateral to medial, connecting #1 to #3 above.

Abbreviations: SP: spinous process; CSA: cross-sectional area; LM: lumbar multifidus; ES: erector spinae
