# Supplementary material for: Assessing lumbar paraspinal muscle cross-sectional area and fat composition with T1 versus T2-weighted magnetic resonance imaging: Reliability and concurrent validity
Source: PLoS One. 2021 Feb 5;16(2):e0244633. doi: 10.1371/journal.pone.0244633 (PMC7864460; doi:10.1371/journal.pone.0244633)
Supplement: S2 File — (PDF) [file pone.0244633.s002.pdf]

1) Each muscle is divided into 4 quadrants, bounded as follows (see example below:
   - Anterior: from the dividing line provided on the image to the point where the muscle outline transitions from “across” to “down” in orientation
   - Lateral: from the anterior-to-lateral ms outline transition point to where the down/across outline transition of the posterior ms outline occurs
   - Posterior: from the lateral-to-posterior ms outline transition to the point where the retrospinous ms/fat transition occurs
   - Medial: from the posterior-to-medial ms outline transition to the medial-to-anterior ms outline transition.
   - **Caveat**: for any transition point above where the two outlines do not overlap, use the most “external” outline margin as the reference point and extend the “dividing line” ~45° to intersect the internal outline margin

2) Alignment scale criteria:
   - Perfect/near perfect: direct overlap of the entire quadrant outline ➡ <2mm gap extending <50% of the quadrant <OR> 2-3mm gap extending ≤10% of the quadrant [Note: direct overlap = no clear gap between the lines]
   - Mild mismatch: 1mm gap ≥50% of the quadrant <OR> 2-3mm gap >10% ➡ no part of the gap is ≥4mm over 10% of the quadrant
   - Significant mismatch: ≥3mm gap extending ≥50% of the quadrant outline <OR> any gap ≥4mm extending >10% of the quadrant
   - **Caveat 1:** if a mismatch occurs across a quadrant transition, assess each quadrant based on the “dividing line” used under section 1’s caveat guideline.
   - **Caveat 2:** gap % = total amount of combined gaps; gap width = maximum width at any point along the quadrant. When determining gap % base on width criteria, include the entire gap length total, not just the gap that falls within a particular width (e.g., not just the part of the gap over 3mm, but the whole gap).

3) To ensure equivalent measurements between assessors, document must be set to magnification of 200%, and a 24” (60cm) monitor used.

4) Once assessment is completed individually, each set of images will be reviewed by both assessors to reach consensus on the final scale.

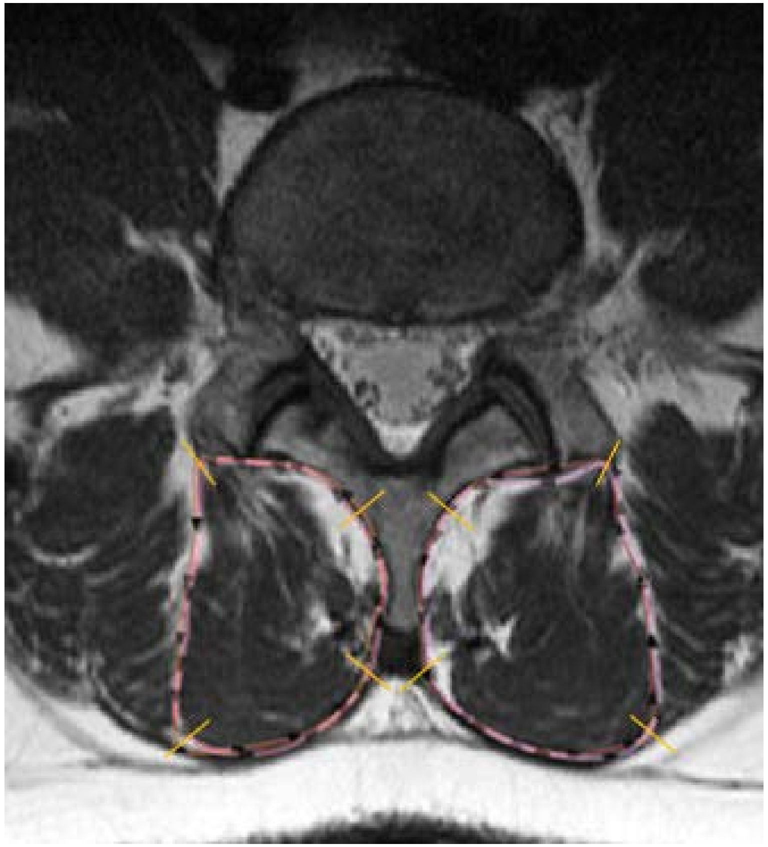

Example marking sheet: compares overlapped T1 and T2 images of the same slice.

Case # ⬡

T1/T2 Outline Comparison: L4/L5

Images to compare
[set screen magnification to 200%]

Right

☐ Ant

☐ Lat ☐ Med

☐ Post

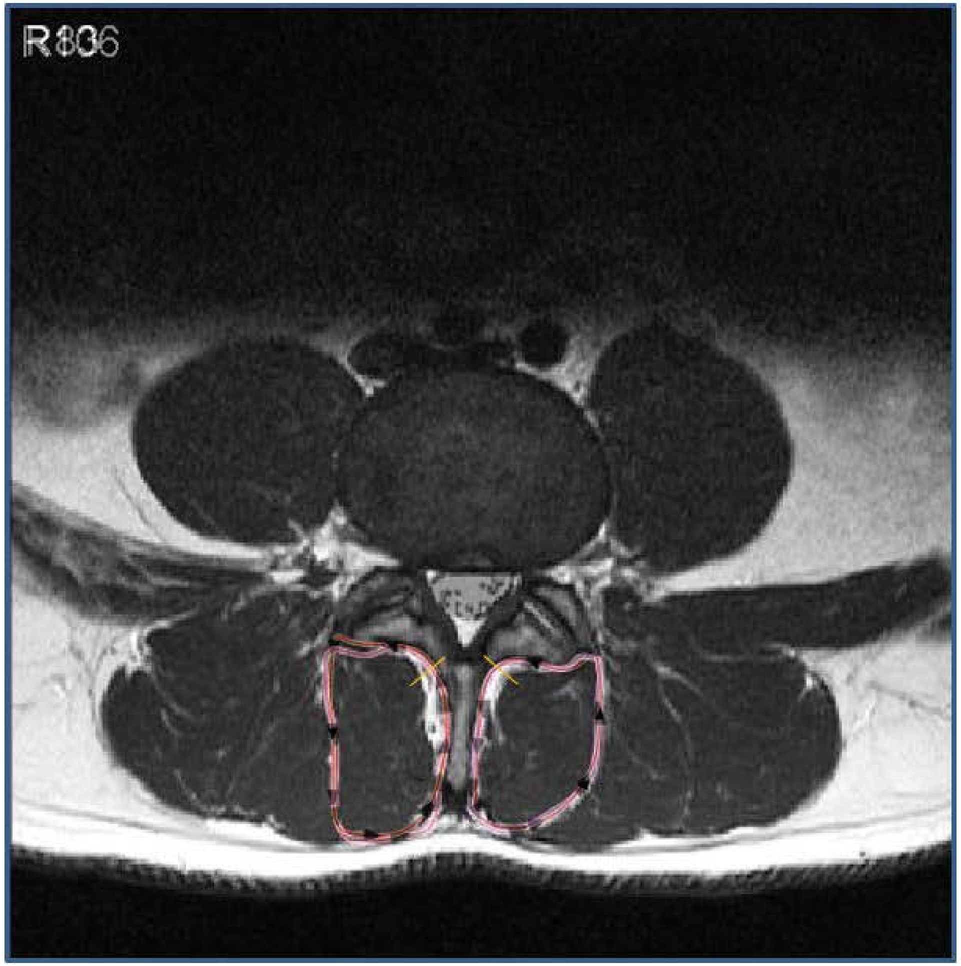

Left

☐ Ant

☐ Med ☐ Lat

☐ Post

Muscle outline alignment scale: 0 = perfect/near perfect; 1 = mild mismatch; 2 = significant mismatch
