# Supplementary material for: Assessing lumbar paraspinal muscle cross-sectional area and fat composition with T1 versus T2-weighted magnetic resonance imaging: Reliability and concurrent validity
Source: PLoS One. 2021 Feb 5;16(2):e0244633. doi: 10.1371/journal.pone.0244633 (PMC7864460; doi:10.1371/journal.pone.0244633)
Supplement: S3 File — (PDF) [file pone.0244633.s003.pdf]

```
*****************************************************************************************************************************
**** Program blandaltman (one parameter against another parameter) startet *********************************************
*****************************************************************************************************************************

    set more off
    capture program drop blandaltman
    program blandaltman
        syntax varlist(max=2)

        // prepare for Bland Altman Interreader
            tempvar diff_xy
            tempvar avg_xy
            tempvar lower
            tempvar higher
            tempvar MW
            tempvar SE
            tempvar MOVERLower  // for sqrt calulation in MOVER method for lower LOA CI calculations (3.8416 directly substituted for 1.96^2) //
            tempvar MOVERUpper  // for sqrt calulation in MOVER method for upper LOA CI calculations //
            tempvar CHILower    // "(a-1^2)" calculation in MOVER formula //
            tempvar CHIUpper    // "(b-1^2)" calculation in MOVER formula //
            tempvar CHIDIFFLower    // to calcuate "a" in MOVER formula //
            tempvar CHIDIFFUpper   // to calcuate "b" in MOVER formula //
            tempvar CIhigher
            tempvar CIlower
            tempvar UpperLOACIhigher   // final calculation to determine upper CI for the upper LOA //
            tempvar UpperLOACIlower   // final calculation to determine lower CI for the upper LOA //
            tempvar LowerLOACIhigher   // final calculation to determine upper CI for the lower LOA //
            tempvar LowerLOACIlower   // final calculation to determine lower CI for the lower LOA //

            generate `diff_xy'=0
            generate `avg_xy'=0
            generate `lower'=0
            generate `higher'=0
            generate `MW'=0
            generate `SE'=0
            generate `MOVERLower'=0
            generate `MOVERUpper'=0
            generate `CHILower'=0
            generate `CHIUpper'=0
            generate `CHIDIFFLower'=0
            generate `CHIDIFFUpper'=0
            generate `CIhigher'=0
            generate `CIlower'=0
            generate `UpperLOACIhigher'=0
            generate `UpperLOACIlower'=0
            generate `LowerLOACIhigher'=0
            generate `LowerLOACIlower'=0

        // count the variable: how many variable are in the list?
            local noofvars : word count `varlist'
            display as text "The variable list of this program counts " `noofvars' " variables"
            display as result " "
            display as result " "
```

```
    // Interreader
        local x = 1
        local y = 1
        foreach varx of varlist `varlist' {
            foreach vary of varlist `varlist'{
                if `y' >`x'{
                quietly replace `avg_xy'=(`varx'+`vary')/2
                quietly replace `diff_xy'=`varx'-`vary'
                display as result "Bland Altman Plot of `varx' and `vary'"
                quietly sum `diff_xy'
                quietly return list
                quietly replace `MW'=r(mean)
                quietly replace `lower'=r(mean)-1.96*r(sd)
                quietly replace `higher'=r(mean)+1.96*r(sd)
                quietly replace `SE'=(r(sd))/(sqrt(r(N)))
                quietly replace `CHIDIFFLower'=sqrt((r(N)-1)/invchi2tail(r(N)-1,0.025))
                quietly replace `CHIDIFFUpper'=sqrt((r(N)-1)/invchi2tail(r(N)-1,0.975))
                quietly replace `CHILower'=(`CHIDIFFLower'-1)*(`CHIDIFFLower'-1)
                quietly replace `CHIUpper'=(`CHIDIFFUpper'-1)*(`CHIDIFFUpper'-1)
                quietly replace `MOVERLower'=sqrt((3.8416/r(N))+(3.8416*`CHILower'))
                quietly replace `MOVERUpper'=sqrt((3.8416/r(N))+(3.8416*`CHIUpper'))
                quietly replace `CIlower'=r(mean)-1.96*`SE'
                quietly replace `CIhigher'=r(mean)+1.96*`SE'
                quietly replace `UpperLOACIlower'=r(mean)+1.96*r(sd)-(r(sd)*`MOVERLower')
                quietly replace `UpperLOACIhigher'=r(mean)+1.96*r(sd)+(r(sd)*`MOVERUpper')
                quietly replace `LowerLOACIlower'=r(mean)-1.96*r(sd)-(r(sd)*`MOVERLower')
                quietly replace `LowerLOACIhigher'=r(mean)-1.96*r(sd)+(r(sd)*`MOVERUpper')
                display as result "Mean difference: " `MW' " (CI "  `CIlower' " to "  `CIhigher' ")"
                display as result "sd of difference between `varx' and `vary' is "r(sd)
                display as result "Upper Limits of agreement: "  `higher' " (CI "  `UpperLOACIlower' " to " `UpperLOACIhigher' ")"
                display as result "Lower limits of agreement: "  `lower' " (CI "  `LowerLOACIlower' " to " `LowerLOACIhigher' ")"
                display as result "Max mean of differences between `varx' and `vary' is "r(max)
                display as result "Min mean of differences between `varx' and `vary' is "r(min)
                display as result "N="r(N)

                label var `diff_xy' "Values"
                label var `MW' "mean of difference"
                label var `lower' "lower limit of agreement"
                label var `higher' "higher limit of agreement"
                label var `UpperLOACIlower' "lower limit of upper agreement"
                label var `UpperLOACIhigher' "higher limit of upper agreement"
                label var `LowerLOACIlower' "lower limit of lower agreement"
                label var `LowerLOACIhigher' "higher limit of lower agreement"
                twoway (scatter `diff_xy' `avg_xy', msymbol(smdiamond_hollow) mcolor(cranberry))(line `MW' `avg_xy', lwidth(medthin) lcolor(blue))
                  (line `CIlower' `avg_xy', lwidth(vthin) lcolor(midblue))(line `CIhigher' `avg_xy', lwidth(vthin) lcolor(midblue))
                  (line `lower' `avg_xy', lwidth(medthin) lcolor(black) )(line `higher' `avg_xy', lwidth(medthin) lcolor(black) )
                  (line `UpperLOACIlower' `avg_xy', lwidth(vthin) lcolor(green) )(line `UpperLOACIhigher' `avg_xy', lwidth(vthin) lcolor(green) )
                  (line `LowerLOACIlower' `avg_xy', lwidth(vthin) lcolor(green) )(line `LowerLOACIhigher' `avg_xy', lwidth(vthin) lcolor(green) ),
                   title(  ) subtitle() xtitle(Average of `varx' and `vary', size(3)) ytitle(Difference of `varx' and `vary', size(3)) caption() note(Bilateral)  legend(off)
                }
            local y = `y'+1
            }
        local y = 1
        local x =`x'+1
        }
    end

*******************************************************************************************************************************
**** Program blandaltman (one parameter against another parameter) endet ********************************************************
*******************************************************************************************************************************
```
